# Complex intervention for MS patients in Germany – can it be cost-saving for the society?

**DOI:** 10.1007/s10198-026-01907-6

**Published:** 2026-03-04

**Authors:** Markus Krohn, Susan Raths, Steffen Fleßa

**Affiliations:** Lehrstuhl für Allgemeine Betriebswirtschaftslehre und Gesundheitsmanagement, Rechts- und Staatswissenschaftliche Fakultät, Universität Greifswald, Friedrich-Loeffler-Str. 70, D-17487 Greifswald, Germany

**Keywords:** Economics of neurology, Cost of MS, Life-time cost, MSnetWork, Multiple sclerosis, System dynamics

## Abstract

**Background:**

Multiple sclerosis (MS) is a chronic degenerative neurological disease with high prevalence and economic impact on the patient and the society. The innovative care project MSnetWork provides services additional to the standard care of MS patients in order to reduce cost of sickness days. This paper calculates the life-time cost of MS in Germany and compares them with the intervention cost MSnetWork in order to determine whether that intervention can be cost-saving.

**Methods:**

We calculated the process cost of the intervention of MSnetWork. Furthermore, we developed a system dynamics model to determine the life-time costs of MS from a societal perspective, both as a present value (at age of onset) and as an annuity. We compare the total life-time cost with and without intervention in order to determine whether MSnetWork can be cost-saving.

**Results:**

The average cost of implementing all measures of MSnetWork was EUR 1212.83 (SD = 692.34) per patient per year. Based on the life-time cost model, a patient with MS costs about 21,000€ more per year than a comparable member of the statutory health insurance fund without MS. The indirect cost is higher than the direct cost. The interventions of MSnetWork reduce the indirect cost substantially.

**Conclusions:**

The economic cost of MS is high. Thus, interventions to reduce disability can certainly be cost-saving. However, an intervention cannot be considered cost-saving per se. It has to be planned precisely and in detail. The results indicate that the MSnetWork intervention can be cost-saving.

**Supplementary Information:**

The online version contains supplementary material available at https://doi.org/10.1007/s10198-026-01907-6.

## Background

Multiple sclerosis (MS) is a chronic degenerative neurological disease with high prevalence and disease burden. The Institute for Health Metrics and Evaluation (IHME) estimates that approximately 2.04 million people worldwide had MS in 2023, resulting in a loss of 1.07 million disability-adjusted life years (DALYs) [1], although these figures are likely to underestimate the true situation as some population groups (e.g. Black and Hispanic in USA [2]) and in particular patients in low-income countries are not sufficiently covered [3]. In Germany alone, more than 250,000 people are living with the disease [4], with an incidence of 13,400 new cases per year [5]. As multiple sclerosis is not yet curable, but is no longer associated with a significantly reduced life expectancy, the prevalence is steadily increasing [6].

The economic impact of MS is considerable. Kobelt et al. [7] show that the average cost per case and year in Europe is €22,800 (mild course), €37,100 (moderate course) and €57,500 (severe course). Treatment costs account for 68% of the costs in mild cases, 47% in moderate cases and 26% in severe cases. To assess the efficiency of an intervention (e.g. against premature disability), it is necessary to compare the cost of the intervention with the cost of remaining life expectancy. However, most studies only calculate costs for a specific year, without considering the life-time costs of patients. For example, Pike et al. analysed 80 studies on the cost of MS, none of which calculated life-time costs [8], so no statements can be made about the long-term efficiency of interventions. As the improved treatment will have long-term benefits, it is necessary to analyse the life-time and not only the length of a project to assess its potential for improved and efficient healthcare [9].

As shown in mode details in the next section, the MSnetWork Innovation Fund project implements a new form of care and aims to maintain or even improve the health of people with MS and positively influence their ability to work and participate in social life [10]. A network of neurologists, occupational and rehabilitation physicians, psychologists and social insurance agencies provides patients with targeted, primarily social-medical services that were not previously part of standard care. Treatment options are identified and interventions initiated according to the needs of the individual patient. However, it is unclear whether these additional interventions will reduce the indirect cost due to sickness days, unemployment or early retirement so that the total cost of the intervention, other direct and indirect costs are lower under MSnetWork than for routine care. Thus, we would like to determine the conditions under which the intervention can be cost-saving.

In the following, we calculate the cost of the MSnetWork intervention. Furthermore, a system dynamics model is developed to determine the life-time costs from a societal perspective, both as a present value and as an annuity, which can be compared to the intervention costs in order to assess whether the intervention can be cost-saving.

## Methods

The main objective of this paper is the comparison of the life-time cost of MS with and without this specific intervention. Consequently, we first explain the setting of MSnetWork (section 2.1) and demonstrate the methodology to calculate the process cost of the intervention. As these intervention costs are uncertain, we develop a Monte-Carlo simulation to determine the distribution of intervention cost. Afterwards, we develop a system dynamics model to calculate the cost of MS with and without the MSnetWork intervention from the onset of the disease to the year of death of the patient (section 2.3). Finally, we compare the life-time cost of MS with and without the innovative intervention including the intervention cost from a societal perspective (section 2.4). Generally, in this paper we will use the term “intervention” when we refer to the specific elements of MSnetWork beyond the standard care. All counselling and treatment offered by the neurological practices will be referred to as healthcare services including standard care. The entire treatment process can be divided into several sub-processes. One of them can be the MSnetWork intervention.

### Setting: MSnetWork

MSnetWork is a new approach to care in Germany’s ambulatory setting. It was implemented from 2022 to 2025 and financed by the German Innovation Fund, a fund introduced by the German Government to finance innovative complex intervention enriching standard care in Germany in order to test whether they could become an element of standard care in future. The project’s primary objective was to evaluate the effectiveness of interventions in enhancing the employment prospects and social participation of MS patients. To this end, a network of neurologists, occupational and rehabilitation physicians, psychologists and social insurance providers came together to offer innovative services to patients that are not part of routine care in Germany. All elements of routine care were continued, the components of MSnetWork came additionally on top of the routine care package. These services included enhanced, participatory consultations with neurologists, occupational health examinations with occupational physicians, psychosocial and legal counselling with qualified professionals, disease-specific education with neuropsychologists or neurologists, and MS-specific rehabilitation with rehabilitation physician [10]. This approach aims to identify and mitigate the risk of sickness absence, unemployment, and early retirement.

Based on the assessment of the neurologist and occupational physician, the following options can be selected to improve workability:

- occupational health examinations to determine stress factors associated with sickness absence and find ways to overcome them.
- Disease education to develop coping strategies with and for the patient, particularly increasing the patient’s sense of responsibility for their situation, including their employment situation.
- Rehabilitation in a specialised rehabilitation centre to stabilise health and improve workability.
- Psychosocial and legal counselling to improve social and employment stress.

These interventions are not part of routine care and aim to improve the health of MS patients so they can return to work, continue working, reduce sickness absence and avoid premature retirement.

Patients were randomly assigned to a control (*N* = 40) and an intervention group (*N* = 60), respective data was retrieved from the health insurance funds and by questionnaires. The primary outcome of MSnetWork is the number of days of sick leave associated with MS. Quality of life data is collected in a parallel project, but this is not included in the results presented here and will have to be analysed elsewhere.

### Intervention cost

We followed a standard procedure of activity-based costing to calculate the cost of the MSnetWork intervention per patient per year. This concept of activity-based costing is widely used in business to calculate the cost of processes and products, in particular in the service industry with high overheads [11]. It consists of the steps process analysis, resource analysis and cost analysis resulting in an estimate of process or unit costs which is more precise than for other costing instruments. It is also applied to calculate the cost of health care interventions [12, 13] because healthcare services usually have low variable cost. Activity-based costing allows to allocate the overheads rather precisely to different processes producing more precise results than other instruments. For scientific purposes, the three steps are enriched by a Monte-Carlo simulation in order to deal with uncertainty and produce a distribution of process or unit cost.

- Process analysis: A process chart was developed based on the intervention design. Afterwards, a process workshop was conducted with four neurological practices, one occupational health physician and representatives of the Board of Neurology Germany. We presented the process charts prepared by the scientists based on guidelines of the German Board of Neurology and the MSnetWork project proposal. Each step of the process was discussed. As the number of experts was small, consensus was reached rather easily. However, in some cases it was realized that the processes differ between neurological practices in particular if they do not comply with the official regulations or guidelines. In this case, we followed the official guidelines. At the end of the day, there was an agreed-on standard process chart.
- Resource analysis: We determined relevant resources consumed in each sub-process, in particular working time of neurologists and assistants, but also materials consumed. Based on a questionnaire, staff of neurological practices assessed the consumption of materials and time per sub-process.
- Cost analysis: We valued each resource unit (e.g. cost per working minute of a neurologist, cost of working minute of an assistant) with their full cost. This step includes a frequency analysis, i.e., how often each process is performed per year for an individual patient.
- Monte-Carlo simulation: The total costs per patient per year were determined using Monte Carlo simulation. This simulation was introduced in order to analyze the impact of different workload on the unit cost (e.g. time consumed per patient and process or number of patients in the physician’s office). We did a distribution identification with Minitab Statistical Software (Fa. Minitab GmbH), and added border values for minimum and maximum. Finally, we repeated the simulation with 10,000 runs. In this way, the Monte-Carlo simulation deals with uncertainty of parameters and allows probabilistic statements about costs [14, 15] The results of the distribution identification and the calculation steps of the simulation can be found in the attachment (Att. 1).

### Life-time cost

We developed a system dynamics model to calculate the life-time cost of MS that allows a comparison of the present value of the total cost and the respective annuity with and without the interventions of MSnetWork. The present value is the current value of a future stream of costs (or utilities), while the annuity is the average cost per year including compound interest. System dynamics models are basically systems of simultaneous difference equations permitting the calculation of time series with feedback loops [16, 17].

The model calculates the population of sex *s* (where *s = 1*: male, *s = 2*: female) with MS in year of life *a* (*P*_*sa*_), on the condition that the disease occurs at age *e*. At age *e*, the number of new cases *E* is added, where *g* is the proportion of women. As the model does not determine the budgetary impact of MS but the life-time cost, it is useful to add up all patients at a given age *e*, with different scenarios of *e* being calculated in the analysis. The following therefore applies:

$${P}_{1e}=\left(1-g\right)\bullet E\ resp..{P}_{2e}=g\bullet E$$

Ageing is performed annually, taking into account the sex- and age-specific mortality of MS patients with sex *s* at age *a (m*_*sa*_*)*. Disease-, sex- and age-specific mortality is calculated by multiplying the sex- and age-specific mortality rate by the standardised disease-specific mortality rate (*SMR*). This results in:

$${P}_{sa+1}={P}_{sa}\left(1-{m}_{sa}\right)\ \textrm{for}\ s=\textrm{1..2};a=e..100$$

The direct costs (*C* _ *d*_*sa*_) result from the annual treatment cost of a patient (*c*_*sa*_) multiplied by the number of patients in gender *s* and age group *a*. The indirect costs of MS (*C* _ *i*_*sa*_) are calculated according to the human capital approach from the number of patients in gender *s* and age *a* multiplied by the specific labour force participation rate *w*_*s*a_ and the gender- and age-specific gross wage costs per year (*S*_*sa*_), which are increased by non-wage labour cost factor (*l*_*sa*_). In addition, the MS-related days of incapacity to work are valued in monetary terms. For this purpose, the days of incapacity to work (*AU*_*sa*_) are weighted by the cost per working day, with the model assuming an average of 220 working days per year. This results in:

$$C\_{d}_{sa}={P}_{sa}\bullet {c}_{sa}\ resp.$$

$$C\_{i}_{sa}={P}_{sa}\bullet {w}_{sa}\bullet {S}_{sa}\bullet \left(1+{l}_{sa}\right)+{P}_{sa}\bullet {AU}_{sa}\bullet \frac{S_{sa}\bullet \left(1+{l}_{sa}\right)}{220}\kern0.5em \textrm{for}\ s=\textrm{1..2};a=e..100$$

The labour force participation rate (*w*_*sa*_) considers sickness, age and gender-specific rates for full-time and part-time employment. The model assumes a 50% reduction in hours worked for part-time employment.

Table 1 shows the parameters used.

**Table 1:** Parameters

*P*_*sa*_	Number of patients with sex *s* in year of life *a*, s∈{male, female} *a* = *e*…100
*e*	Age of onset
*E*	Number of incident cases per year
*g*	Proportion of women among incident MS patients
*m*_*sa*_	Mortality of MS patients with sex *s* at age *a*, s∈{male, female}, *a* = *e*…100
*SMR*	Standardised disease-specific mortality index
*w*_*sa*_	Labour force participation rate by sex *s* and age *a*, s∈{male, female}, *a* = *e*…100
*S*_*sa*_	Gross wage per annum of an employee of sex *s* and age *a*, s∈{male, female}, *a* = *e*…100
*l*_*sa*_	Non-wage labour costs (factor) of an employee of sex *s* and age *a*, s∈{male, female}, *a* = *e*…100
*C_d*_*sa*_	Direct costs of all patients of sex *s* and age *a*, s∈{male, female}, *a* = *e*…100
*C_i*_*sa*_	Indirect costs of all patients of sex *s* and age *a*, s∈{male, female}, *a* = *e*…100
*c*_*sa*_	Treatment costs per year for an insured of sex *s* and age *a*, s∈{male, female}, *a* = *e*…100
*AU*_*sa*_	Days of incapacity to work per year for a patient of sex *s* and age *a*, s∈{male, female}, *a* = *e*…100
*i*	Discount rate
*LE*	Life expectancy
*B*_*C*_	Present value of life-time costs
*A*_*C*_	Annuity of life-time costs

The present value of the life-time costs (*B*_*C*_) per MS patient is therefore calculated as

$${B}_C=\frac{\sum_{a=e}^{100}\sum_{s=1}^2\left(C\_{d}_{sa}+C\_{i}_{sa}\right)\bullet {\left(1+\frac{i}{100}\right)}^{e-a}}{E}$$

The specific life expectancy (*LE*) must be determined for the annuity. To do this, the number of deaths in the total population (men and women) is weighted by age at death and the arithmetic mean is calculated for all years, i.e.

$$LE=\frac{\sum_{a=e}^{100}a\bullet \left({P}_{1a}\bullet {m}_{1a}+{P}_{2a}\bullet {m}_{2a}\right)}{E}$$

The annuity (*A*_*C*_) thus corresponds to the cost per year from the age of onset (*e*) to the expected time of death. Men and women are weighted according to their share *g* in the calculation of average life expectancy. This gives the annuity as

$${A}_C={B}_C\frac{q^{LE-e+1}\bullet \left(q-1\right)}{q^{LE-e+1}-1}\ with\ q=1+i$$

Table 2 shows the parameters taken from the literature.

**Table 2:** Parameters und values (SHI = Statutory health insurance, PHI: private health insurance)

Parameter	Explanation	Value(s)	Source
*e*	Age of onset	32; 43	Flachenecker et al. [18], Graf et al. [5])
*E*	Number of incident cases per year (SHI + PHI)	13,400	Graf et al. [5]
*g*	Proportion of women among incident MS patients	70%	Petersen et al. [19]
*m*_*sa*_	Mortality of MS patients with sex *s* at age *a*, *s*∈{male, female}, a = e…100	Mortality table	Destatis [20–22]
*SMR*	Standardised disease-specific mortality index	1; 2.7	Lunde et al. [23] Smyrke et al. [24]
*w*_*sa*_	Labour force participation rate by sex *s* and age *a*, s∈{male, female}, a = 20…100	various	Flachenecker et al. [18], Demographieportal [25])
*S*_*sa*_	Gross wage per annum of an employee of sex *s* and age *a*, *s*∈{male, female}, *a* = 20…100	various	Demographieportal [25]
*l*_*sa*_	Non-wage labour costs (factor) of an employee of sex *s* and age *a*, *s*∈{male, female}, *a* = *e*…100	23.3%	Destatis [20–22]
*c*_*sa*_	Treatment costs per year for an insured of sex *s* and age *a*, *s*∈{male, female}, *a* = *e*…100	MS: € 18.928,82 Average insured person: € 4.123,25	Müller et al. [26], BMG [27]
*AU*_*sa*_	Days of incapacity to work per year for a patient of sex *s* and age *a*, *s*∈{male, female}, *a* = *e*…100	various	([28], [20–22])
*i*	Discount rate/interest rate	0.05 (5%)	

The age of onset is given as 43 years by Graf et al., based on data from health insurance companies [5], while Flachenecker et al., based on data from the MS registry, estimate it to be 32years [18]. The standardised disease-specific mortality index was originally estimated at 2.7 [24], while it is nowadays generally accepted that excess mortality no longer occurs [23]. Accordingly, different scenarios need to be calculated for the age of onset and the SMR.

Treatment costs for an average insured person in Germany amount to €4123.25 [27]. The MS-specific treatment costs of an MS patient from the publication by Müller et al. [26] were compounded to 2023. This results in direct costs for MS patients of €23,052.07. Due to unavailability of data, the following calculations are simplified using a constant value for treatment costs independent of gender and age and the course of disease. Discounting is carried out at a rate of 5% p.a [29]..

The cost per patient in MSnetWork is calculated as the sum of the project-specific individual service payments per year. The maximum scope of care includes the sub-interventions listed in Table 3.

**Table 3:** Sub-interventions incl. Frequency of implementation per year and prices per service and patient. Source: Own

Sub-intervention	Frequency p.a.	Price per service and patient [€]
Quarterly consultation, incl. Coordination	4	65
Functional assessment	2	45
Psychosocial and socio-legal counselling	1	75
Neuroeducation module	1	228–455 (depending on the number of participants)
Occupational health care	1	190
Teleconsultation	1	30
Case conference	1	60

The implementation of all intervention services results in a total cost of approximately €1000 per patient per year. This assumes that the interventions are carried out with the same frequency and intensity each year as the disease progresses.

### Cost-saving

We calculate the present value (and annuity) of the life-time cost with and without the interventions of MSnetWork. The life-time cost includes the intervention cost as well as the other health service cost as direct cost and the indirect cost due to sickness days and pre-mature retirement. It is self-evident that the calculation of the life-time cost with intervention includes the intervention cost calculated according to section 2.2. In a health economic perspective, any intervention can have four different results:

1. Increase utility and increase (life-time) cost
2. Increase utility and reduce (life-time) cost
3. Decrease utility and increase (life-time) cost
4. Decrease utility and reduce (life-time) cost

It is obvious that (2) is the best and (3) the worst situation, but (1) and partly even (4) might be of interest for further analysis or even decision-making. In the case of MSnetWork, no health outcomes are known (yet). However, the interventions of MSnetWork are additional to standard care, i.e., no elements of standard care are omitted in order to implement MSnetWork. Consequently, it can be assumed that the utility for the patient with MSnetWork is at least as high as without MSnetWork. If MSnetWork had substituted some elements of routine care, we might challenge this assumption. But as MSnetWork simply adds to routine care, there is good reason to assume that the utility of patients with MSnetWork is not less than the utility of patients without MSnetWork.

Under this condition, it is worthwhile to study the life-time cost in order to know whether an intervention could have a positive impact on the resources consumed. For instance, if the intervention of MSnetWork reduces the life-time cost of MS, it is worthwhile to consider this intervention even if no traditional outcome measures such as mortality or quality of life are affected. The analysis whether an intervention is cost-saving requires the comparison of the life-time cost with and without the intervention in order to guide decision-makers whether further implantation is helpful. In our case, the main outcome parameter are the sickness days. The respective data is provided by the German sickness fund AOK and entered into the life-time cost calculation.

## Results

### Intervention cost

The Figure in the attachment (Att. 2) shows the process chart of the MSnetWork intervention. Table 4 shows the actual costs measured in the MSnetWork practices. The patient-fixed cost cover cost of preparation, training on neuro-education as well as psycho-social and legal consultation. They amount to €293.08 per patient. The variable cost per intervention per patient and year depend on the frequency and the time consumption and range from €14 (case conference) and €240 (quarterly consultation). Neuroeducation had a very high deviation as it was performed as group consultation with 4–8 participants. In total, the average cost per patient per year were €1212.83 (SD €692.34) with a range between €394.93 and €7949.85. 95% of patients incurred less than €2346.

**Table 4:** Cost of sub-processes of neurological care per patient and year [€]. Source. Own

Process	Average	SD	MIN	MAX	95. percentile
Project preparation	110.53	155.86	1.25	2323.20	381.61
Quarterly consultation	238.15	95.99	51.01	628.00	433.24
Functional assessment	107.96	40.86	25.19	278.67	181.31
Training of staff, neuro-education	161.65	234.30	1.97	2635.20	519.96
Neuro-education	478.70	343.93	149.61	3988.23	967.60
Training of staff, Counselling	20.90	27.59	0.36	283.20	70.80
Counselling	26.46	17.87	1.53	70.80	70.80
Tele-consultation	54.58	39.95	0.00	146.12	121.43
Case conference	13.91	13.98	0.00	36.49	32.62
Total cost	1212.83	692.34	394.93	7949.85	2345.85

Based on the actual cost we simulated the standard cost under the assumption of equally distributed patient numbers of between one and 600 patients per practice. The average standard cost is €876.97 (SD = €271.63) per patient with an evenly distributed number of patients. If the number of patients is less than six, the individual costs per patient are almost twice as high at €1596.68 (SD = €882.89). For ten or more patients treated, the average individual costs are €847.97 (SD = €22.23) per patient.

As expected, high unit costs occur if few patients are treated in the practice, and fluctuations in the individual care time are more relevant if only few patients are cared for. The main difference is due to the degression of overheads. For the care of ten patients per practice, the overhead cost due to the necessary training (introduction, neuroeducation and psychosocial and socio-legal counseling) is €219.97 compared to €43.59 and €23.76 for 50 and 100 patients respectively.

### Life-time cost

The model calculates the present value of the additional disease-specific costs over the life-time of a person with MS. This is achieved by calculating and comparing the costs of a population with and without MS. For a patient without MS, the present value of direct treatment costs is €37,504.83 or an annuity of €2183.47. The indirect life-time costs due to lost years of work and days off work (not due to MS) are €129,578.83 or an annuity of €7543.86, based on a life expectancy of 81.98 (78.95 men; 83.28 women), calculated for an age *e* = 43. Table 5 also shows the values for a present value with an age *e* = 32. The respective costs of the individual scenarios with MS are compared with this standard.

**Table 5:** Present values of life-time costs of people without MS [€]. Source: Own

Age		Direct cost	Indirect cost	Total
32	per capita	69,996.07	238,546.72	308,542.80
	Annuity	3814.65	13,000.32	16,814.97
	Life expectancy			81.64
43	per capita	37,504.83	129,578.83	167,083.65
	Annuity	2183.47	7543.86	9727.32
	Life expectancy			81.98

Table 6 shows the additional costs and years of life lost due to MS. In the standard model with MS, the present value of direct and indirect costs is €316,185.78 higher than in the model without MS, which corresponds to an annuity of €20,934.51 (*e* = 43), i.e. each MS patient incurs an average cost of €20,934.51 per year. 9517.93 is the annuity of treatment costs, the rest is the average loss of productivity due to early retirement and days of incapacity to work. Life expectancy is reduced by 8.63 years. In terms of total costs per person, women and men are almost identical (men: €313,790.77 or €21,022.36 p.a.; women: €317,212.22 or €20,729.52 p.a.), although men have slightly lower direct costs (€134,659.24 or €8731.92 p.a.) because they have a shorter life expectancy (5.25 years with MS, 4.51 years without MS) than women. On the other hand, they have slightly higher indirect costs (men: €179,131.53 or €11,997.60 p.a.; women: €165,031.91 or €11,167.57 p.a.) because the average wage of a man is higher than that of a woman.

**Table 6:** Additional costs and years of life lost to MS in the baseline scenario (e = age of onset). Source: Own

Scenario		Direct cost	Indirect cost	Total
*e* = 32, SMR = 2.7	per capita	294,882.10€	317,813.72€	612,695.82€
	Annuity	17,009.71€	18,752.33€	35,762.04€
	Life expectancy		-€	−9.01
*e* = 43, SMR = 2.7	per capita	146,923.99€	169,261.80€	316,185.78€
	Annuity	9517.93€	11,416.58€	20,934.51€
	Life expectancy			−8.63
*e* = 32, SMR = 1	per capita	321,334.65€	340,765.17€	662,099.82€
	Annuity	17,512.10€	18,571.02€	36,083.12€
	Life expectancy		–	–
*e* = 43, SMR = 1	per capita	172,175.38€	190,457.84€	362,633.22€
	Annuity	10,023.76€	11,088.13€	21,111.88€
	Life expectancy			–

Assuming that mortality is not increased by MS (SMR = 1), no difference in life expectancy is consistently calculated. The present value of direct costs increases slightly, as the increased life expectancy implies higher treatment costs. Indirect costs decrease. However, as the greatest differences in mortality occur at older ages, this does not have a major impact.

If only treatment costs are incurred and mortality and employment do not differ from the normal population, the present value is €172,175.38 and the annuity is €10,034.76. If we consider the case where only reduced earning capacity (sickness days, early retirement) occurs without treatment costs, while mortality increases, the values increase to €190,457.84 and €11,088.13 respectively.

### Cost-saving

Table 7 shows the difference of cost p.a. of loss of working time between control and intervention group. The difference in the arithmetic means is quite high (1044.45 € p.a.); however, the deviation between individuals in the group is quite high as well so that the difference is not significant. Further outcomes are not known (yet) and cannot be considered in this analysis.

**Table 7:** Cost of loss of working time between intervention group (IG) and control group (CG)

Group	N	arithmetic mean [95-% ci]	t-statistics (unpaired t-test)		
			∆ mean [95-% ci]	t-value (df)	*p*-value (2-sided)
IG	227	2005.30 [1368.80 - 2641.80]	− 1033.46 [− 2382.17;315.26]	−1.506 (458)	0.13
CG	233	3038.75 [1856.85–4220.64.85.64]			

However; if we assume that the intervention cost is 1212.83 € per year, the intervention is cost-saving. Due to the fact that MSnetWork merely adds to the routine care, we can assume that the utility of the patient with MSnetWork is not lower than the utility of the patient without MSnetWork. In this case, the intervention also seems to be cost-effective. However, this result depends on the effectiveness of the intervention on employment, i.e. sickness days and early retirement. This is a challenge because this effect is uncertain. Consequently, we have to calculate the threshold of cost-saving behaviour.

**Fig. 1:**
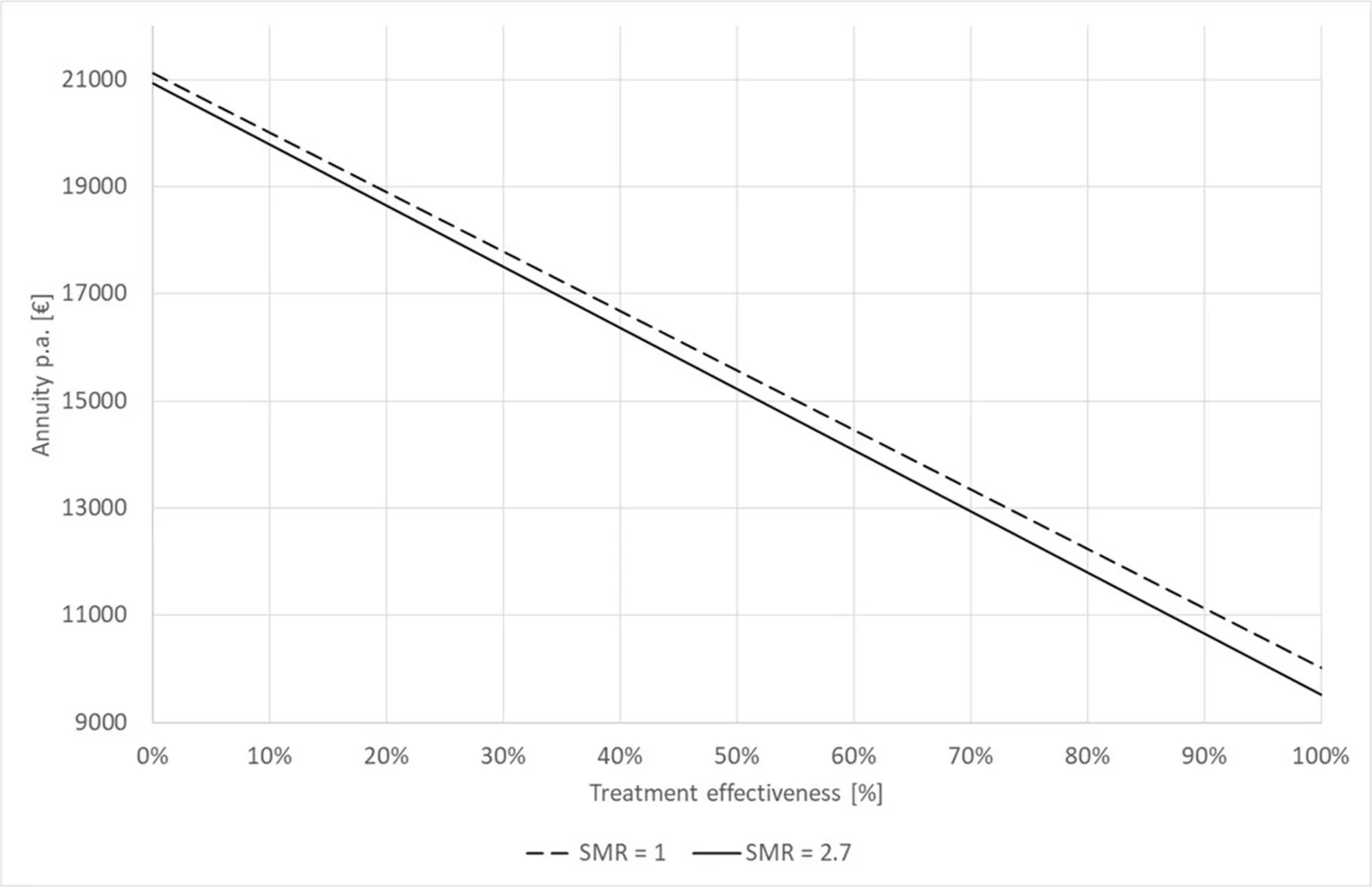
Annuity depending on the success of the treatment. Source: Own

Figure 1 shows the annuity of the additional costs of an MS patient as a function of the effectiveness of treatment on employment (sickness days, early retirement; *e* = 43). For example, if more intensive therapy leads to a 30% reduction in indirect costs, the costs per year fall from €21,111.89 (SMR = 1) or €20,934.51 (SMR = 2.7) to €17,785.45 or €17,509.54, i.e. the intervention would have to cost less than €3326.44 or €3424.97 per year to be cost-saving.

**Fig. 2:**
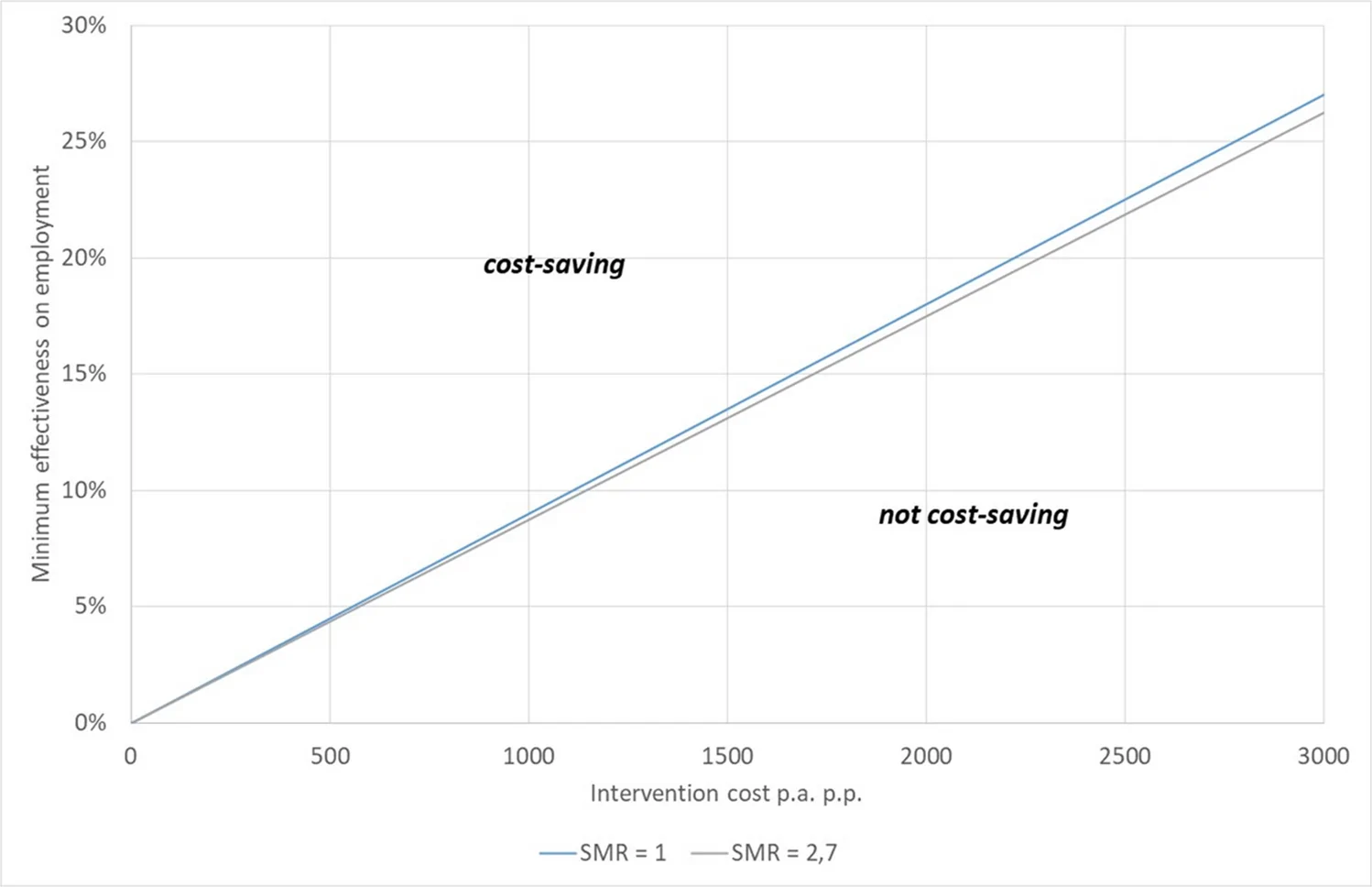
Cost-saving threshold areas depending on intervention cost p.a. and effectiveness of employment. Source: Own

In section 3.1 we showed, that not only the effectiveness of the treatment on employment, but also the intervention cost is uncertain. Thus, it is meaningful to calculate the areas of cost-saving depending on both variables. Figure 2 shows the respective threshold areas. High effectiveness on employment or low intervention costs (area upper left) lead to cost-saving. If, for instance, the treatment cost is as low as 394.93 € (minimum of Table 4), it is sufficient for cost-saving that MSnetWork reduces the loss of employment by 3.6%. If, however, the treatment cost p.a. is as high as 7949.85 (maximum of Table 4), we require an effectiveness on employment on 71.5% (SMR = 1).

## Discussion

### Interpretation of results

The system dynamics model calculates the present value of the life-time costs or annuity of MS patients, with particular emphasis on disease-specific incremental costs. The first question is whether the demographic and epidemiological results of the simulation are reliable. If they differ strongly from the reality, we cannot trust the cost estimates. The second question is how our life-time cost result compares with result from other costing studies. Thirdly, we have to ask whether MS leads to a reduced life expectancy in order to assess the efficiency of the intervention. Fourthly, we will deal with the uncertainty of the intervention cost of MSnetWork by explaining the results of the Monte-Carlo simulation in relation to the life-time costs. The interpretation of results closes with some implication for policy-makers.

Firstly, a demographic model of the total population and of MS patients is used. The German Federal Statistical Office estimates life expectancy at 78.2 years for men and 83.0 years for women [20–22]. The model calculates life expectancy of 78.49 (*e* = 32) and 78.95 (*e* = 43) years for men, 83.00 (*e* = 32) and 83.28 (*e* = 43) years for women and an average of 81.64 (*e* = 32) and 81.98 (*e* = 43) years. The slightly higher values are due to the fact that the Federal Statistical Office gives the life expectancy of newborns, while the model assumes that a person has already survived for 32 or 43 years. The fact that the values for *e* = 32 are lower than for *e* = 43 is also as expected, since the group of patients with a higher age at onset survived 11 years longer. The demographic model is therefore valid.

Secondly, the additional cost findings for MS underline the high overall economic cost of MS. The costs of early retirement and medication account for the largest proportion. The German Federal Office of Social Security (Bundesamt für Soziale Sicherung - BAS) calculated a morbidity surcharge of €13,274 for MS patients on long-term medication for the German risk structure compensation (Risikostrukturausgleich) in 2022, compared to €2798 for MS patients without long-term medication [30]. Compared to Kobelt et al. [7], the calculated annuities of the model are slightly lower. This is partly due to the fact that Kobelt refers to European averages, whereas this model focuses entirely on MS patients from Germany. More importantly, however, the authors do not calculate life-time costs and therefore only record the actual costs for a given year. The model can take this into account by setting the discount rate to 0%. In this case, the annuities are €36,539.83 (*e* = 32) or €36,009.00 (*e* = 43). This corresponds relatively exactly to the moderate (i.e. average) course of Kobelt et al. with an amount of €37,100. In this case, the model calculates a share of 49% (e = 32, e = 43) for the treatment costs, Kobelt et al. 47% (for moderate cases).

Thirdly, a cost-saving analysis has been used before to assess the economic benefit of an intervention, in particular if the outcomes of the intervention are either unknown or at least not inferior to the standard care [31, 32]. The best situation is always if the intervention leads to a better outcome and reduced cost [33] but even without a better outcome the cost-saving potential of the intervention has a value of its own. However, the conditions under which the intervention is cost-saving, must be understood to make wise decisions on introducing the innovative intervention into routine care.

Thus, it is an important question is whether MS leads to reduced life expectancy. As shown, the system dynamics model calculates a difference of 9.01 (*e* = 32) or 8.63 years (*e* = 43) when the SMR is 2.7. In general, the literature still assumes that MS leads to a reduction in life expectancy of up to 10 years [34]. The model calculates a remaining life expectancy after disease onset of 40.63 (*e* = 32) and 30.35 (*e* = 43) years, while the values for the general population are 49.64 (*e* = 32) and 38.98 (*e* = 43) years. Of course, the remaining life expectancy depends on the severity of the disease, the type of MS and secondary diagnoses, but above all on the intensity and professionalism of the treatment. For this reason, the life expectancy of people with MS has increased in recent years, and neurologists now assume that there is no significant reduction in the life expectancy of newly diagnosed people with MS (SMR = 1). However, the calculations showed that this does not lead to completely different results, but to a shift in the cost structure. Patients live longer and therefore incur higher life-time treatment costs, while productivity losses due to mortality decrease.

**Fig. 3:**
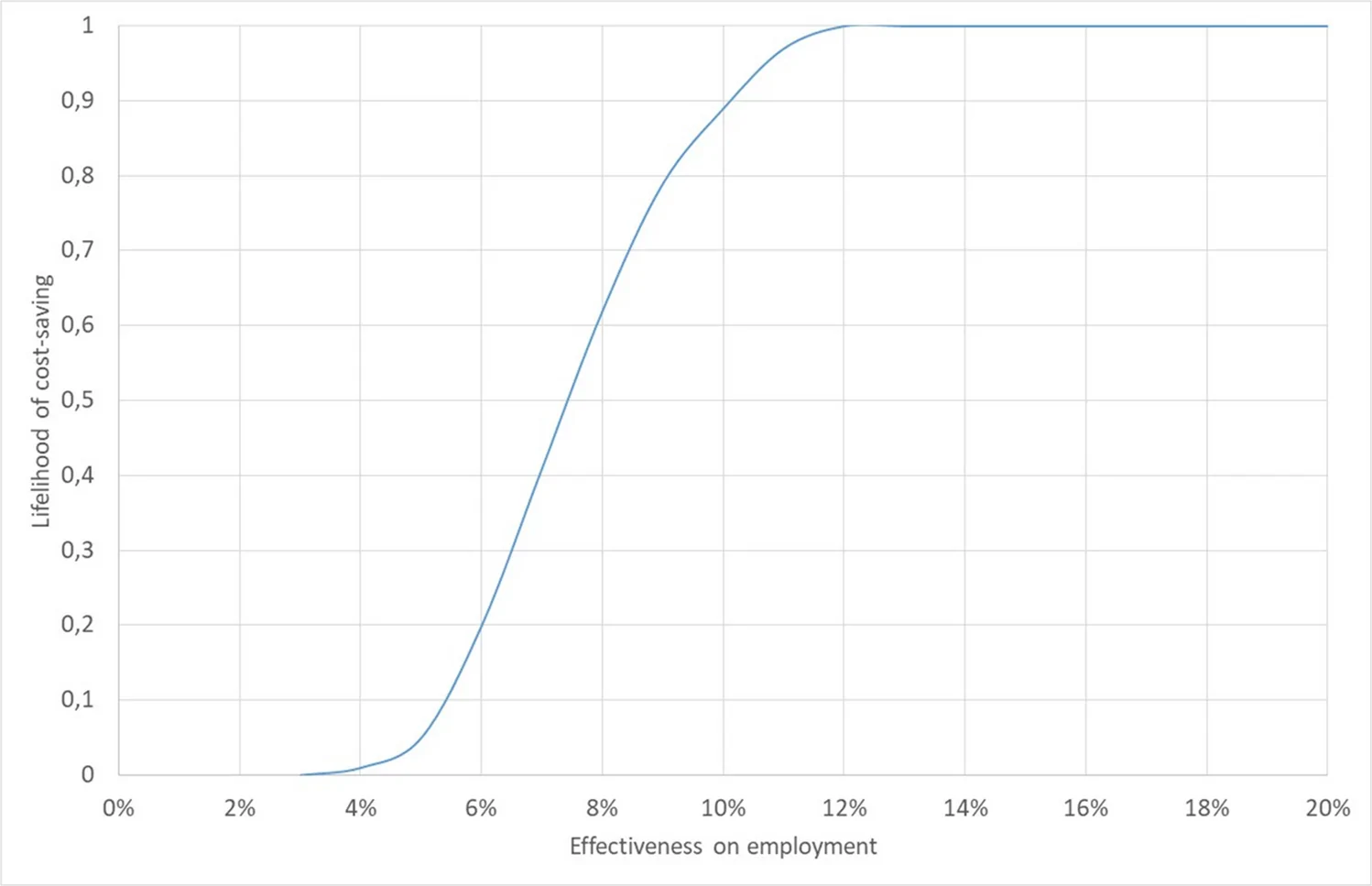
Effectiveness of intervention and likelihood of cost-saving. Source: own

Fourthly, the cost of the complex intervention of MSnetWork has a wide range. As Table 4 demonstrates, they range between 692.34 and 7949.85 Euro. However, the maximum values are rather rare exemption. 50% of cases of intervention cost are equal to or less than 1212.83 Euro, while 95% of interventions have cost of less than 2345.85 Euro. Consequently, it is appropriate to use an estimate of about 1200 Euro as intervention cost for further analyses, but it would be wrong to assume that this figure is always valid and that the intervention is in any case cost-saving. If the minimum actual cost of €394.93 is assumed, the effectiveness of the intervention on employment (i.e. reduction in disability and sickness absence) must be 3.6% so that the intervention is cost-saving. If the arithmetic mean of €1212.83 is assumed, we have to have an effectiveness of 10.9%. For the 95% percentile, the respective value is 21.5%, for the maximum of €7949.85, it is 71.5%. However, the unit cost depends strongly on the number of patients per practice. If we assume that patient numbers are equally distributed, we receive lower unit cost as shown in Fig. 3 attributing the likelihood of being cost-saving to the effectiveness of the intervention on employment. If the patient number is equally distributed, the effectiveness must be at least 7.45% in order to have a 50% likelihood to be cost-saving. We estimate that the MSnetWork intervention will result at least in a reduction of disability and sickness absence of 10% so that we can assess that we have a likelihood of about 48% (for actual cost) or 89% (for standard cost) that the intervention will be cost-saving. Thus, cost-saving is likely, but not guaranteed. Based on discussions with the neurologists (during the process workshop mentioned above), a positive effectiveness of 10% on disability and sickness absence is a conservative estimate, i.e., neurologists are confident that the impact might even be higher. However, there is no proof until today whether this will actually come true.

This analysis is based on the direct and indirect cost as a consequence of our societal perspective. If we take the perspective of the Statutory Health Insurance, an MS-patient is €9517.93 more expensive p.a. (annuity) than a non-MS insured. There is, however, no indication in our data that these direct costs could decline by the complex intervention of MSnetWork. Thus, there is no financial incentive for the Statutory Health Insurance to finance the complex intervention.

For policy-makers, it is crucial to know whether the results of this study are reliable, whether MS has an impact on employability and whether MSnetWork will reduce the indirect cost so that the interventions can become cost-saving. Generally, we can state that the results are quite in line with the findings in the literature and that MS has a major impact on productivity of the patient:

- Share of patients working: Around 60% of MSnetWork patients work at least 35 hours per week, which is essentially full-time. This result is comparable to a survey of MS patients from 2015 [35], in which two-thirds of MS patients stated that they worked full-time.
- Sickness days: The average number of sickness days in the MSnetWork sample is comparable to that described in the literature [36]. report 12.6 and 14.7 sickness days, respectively, for people with at least one sick note per year.
- Sickness cost: Compared to the indirect costs reported by Müller et al. for days of incapacity to work which amounted to €1183 in 2015 (€1380 adjusted for purchasing power), the figures determined in the MSnetWork study are higher at €1760.18 (IG) and 2502.81 (CG) [26]. This difference is due to the different survey and calculation methods and the composition of the total sample. While Müller et al. calculated a flat daily rate of €193 per sickness day and determined sickness days on the basis of routine data, MSnetWork took a more differentiated approach that took into account working time-related and individual income data. In addition, MSnetWork only included patients who were in employment.

The comparison of the control and intervention groups shows that IG patients worked on average 1.3 hours more per week than patients of the CG. However, the differences are not significant. The sickness days in the intervention group were lower than in the CG (12.3 vs. 14.9), i.e., while the number of sickness days in the IG was reduced by 2.2 days, it remained largely constant in the CG (14.8 vs. 14.9 days). However, due to the high proportion of patients without sickness days (approx. 60%) and the large variation in the number of sickness days, the gross number of patients recruited would have had to be higher in order to demonstrate significant effects on sick leave periods. The costs of reduced earning capacity due to a reduction in weekly working hours and days of incapacity for work do not differ significantly between the groups, but the indirect costs in the IG, at €2005.30 per year and patient, are significantly lower than those in the CG (€3038.75). In each case, over 80% of the costs are attributable to sickness days (IG: 87%/CG: 82%). However, this is mainly due to the fact that the majority of patients did not reduce their working hours, with only 26 patients reporting a reduction.

Based on these findings we can conclude that the interventions described in section 2.1 will have a sufficient effectiveness on employment capacity so that the cost of the interventions are covered by a decrease of life-long cost. The loss of working time is more expensive than the intervention. The main problem remains that the intervention has to be paid by the health insurance funds, while the benefit (here: cost-savings) will be earned by the employer (and employees). The split between the institution that has to shoulder the cost and the institution or person who will benefit from the intervention is a challenge not unkown in health economics. It calls for a thorough analysis whether employers could be obliged to pay for certain elements of the program, such as occupational health care. However, this discussion is beyond the scope of this paper.

### Limitations

There are a number of limitations to these analyses that should be taken into account when interpreting the results.

- Design: We developed a control group design assuming that the differences in outcomes (here: sickness days) are due to the MSnetWork interventions. We tried as much as possible to control for other influences but it cannot be guaranteed that there are no random influences beyond our knowledge that challenge causality.
- Calculating sickness days: As employees in Germany can be absent from work for up to three days due to illness without a doctor’s certificate, it is likely that the actual number of sickness days is underestimated. On the other hand, the extent of work incapacity due to MS may be underestimated because only sickness absence with an explicit diagnosis of MS was included in the model. However, it is plausible that there are also days lost due to comorbid or secondary symptoms, such as fatigue or depression, which are at least indirectly attributable to MS. This underlines the importance of accurate reporting of MS-related sickness days for health economic analyses.
- Estimating direct and indirect cost: In addition, the direct costs of illness are likely to be overestimated in the model, as direct costs of illness decrease over time, especially when an initial relapsing-remitting multiple sclerosis (RRMS) changes to secondary progressive multiple sclerosis (SPMS) and the cost of disease-modifying therapy is reduced [37]. On the other hand, MS is a progressive disease which might mean that the assumption of constant intervention cost might underestimate future cost. However, during the project implementation we did not see a major impact of our intervention on the progression. Whether it will be less in coming years due to the intervention, cannot be estimated for the time being. Indirect costs, on the other hand, tend to be underestimated in the model, as the costs of community services and informal care are not taken into account. However, these primarily become relevant at an advanced stage of the disease [37].
- Estimating intervention cost: The calculation of the intervention cost is based on the situation we found in MSnetWork, including the number of patients per practice, the experience and learning level of practice personnel and the intervention intensity during the project. However, the cost of the complex intervention of MSnetWork might decrease if it becomes a routine, i.e., neurologist and assistants might learn to provide the services faster and with lower unit costs. At the same time, the intervention unit cost depends strongly on the number of cases treated. At least in cities, it might be worthwhile to concentrate the intervention in few neurological practices in order to reduce the unit cost. It would also make sense to compare the intervention cost with the quality of life gained by the intervention. However, this data is not available so that we can only estimate cost-saving thresholds, but not cost-effectiveness thresholds. Without doubt, the intervention might be cost-effective even if it is not cost-saving.
- Perspective: Finally, our analysis compares the total life-time cost with the intervention cost of MSnetWork. This is correct from a societal perspective. However, the Statutory Health Insurance Fund has to pay the entire intervention cost (if it becomes a routine), while a major share of the benefits of the intervention are harvested by the society.
- Outcomes: This paper does not report outcomes of MSnetWork as they are not available (yet). We can only prove that the interventions of MSnetWork are cost-saving in most cases (see Fig. 2). For those case where the intervention cost is too high or the effectiveness on employment too low so that the intervention is not cost-saving, it might still be worthwhile making MSnetWork part of the routine care. However, this would require knowledge about health outcomes and calls for further research.
- Uncertainty: As the sample is rather small, there remain quite a degree of uncertainty about the cost of intervention and the effectiveness on employment as the two most crucial variables of the cost-saving threshold. We used sensitivity analyses (see Fig. 1 and Fig. 2) as well as a Monte-Carlo simulation (see Fig. 3) to mitigate the risk of uncertainty, but there is definitely a need to repeat the study with a much higher sample size.

These limitations call for further research, in particular to determine the outcomes of the intervention (e.g. quality of life, mortality). In this way, we could also extent the economic assessment beyond cost-saving and ask whether an intervention might be efficient even if it is not cost-saving.

## Conclusions

Interventions to reduce disability can certainly be cost-saving. However, an intervention cannot be considered cost-effective per se. It has to be planned very precisely and in detail. This shows that the interventions of the MSnetWork project are promising in principle. The cost of the MSnetWork interventions is around €1200 per patient per year if all the individual interventions are implemented. This means that the interventions need to achieve at least a 11% permanent increase in gainful employment (i.e. reduction in disability and sickness absence) in order to be cost-saving. With neuroeducation, psychosocial and socio-legal counselling and occupational health care, the MSnetWork project offers precisely those interventions that appear to have a positive impact on the employment of people with MS. For example, changes to the job or task structure, based on recommendations from the occupational health assessment, can enable people to stay in work or delay retirement. Neuro-educational training can enable patients to cope better with the symptoms of the disease. In addition, psychosocial and socio-legal counselling informs patients about support options, even for the employer, which can have a positive impact on their ability to remain in the workplace.

## Supplementary Information

**ESM 1.**

**ESM 2.**

## Data Availability

All data can be obtained from the authors.

## Abbreviations

BAS: German Federal Office of Social Security (Bundesamt für soziale Sicherung)
DALY: Disability-adjusted life year (lost)
IHME: Institute for Health Metrics and Evaluation
MS: Multiple sclerosis
PHI: Private health insurance
RRMS: Relapsing-remitting multiple sclerosis
SHI: Statutory health insurance
SMR: Standardized disease-specific mortality index
SPMS: Secondary progressive multiple sclerosis

## Declarations

### Authors’ information

SF is professor of healthcare management with a focus on innovative healthcare interventions. SR is post-doc at the department of healthcare management in charge of the economic evaluation of MSnetWork, MK is post-doc at the department of healthcare management.
