# Supplementary material for: Complex intervention for MS patients in Germany – can it be cost-saving for the society?
Source: Eur J Health Econ. 2026 Mar 4;27(7):1549–61. doi: 10.1007/s10198-026-01907-6 (PMC13619654; doi:10.1007/s10198-026-01907-6)
Supplement: Supplementary file 1 [file 10198_2026_1907_MOESM2_ESM.pdf]

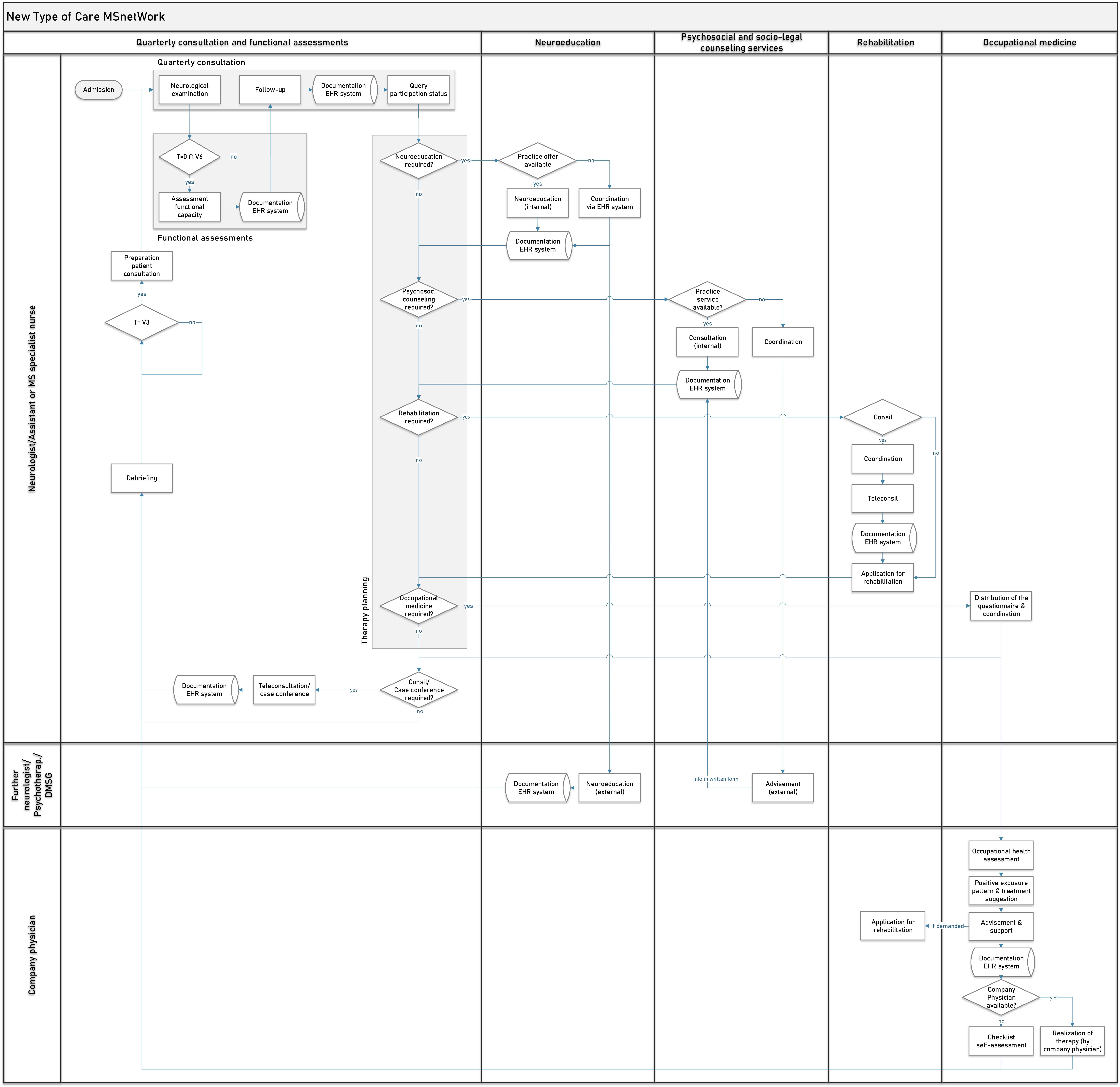

New Type of Care MSnetWork
Quarterly consultation and functional assessments
Neuroeducation
Psychosocial and socio-legal counseling services
Rehabilitation
Occupational medicine
Neurologist/Assistant or MS specialist nurse
Further neurologist/ Psychotherap./ DMSG
Company physician
Quarterly consultation
Admission
Neurological examination
Follow-up
Documentation EHR system
Query participation status
T=0 ∩ V6
no
yes
Assessment functional capacity
Documentation EHR system
Functional assessments
Preparation patient consultation
T= V3
Debriefing
Therapy planning
Neuroeducation required?
Practice offer available
Neuroeducation (internal)
Coordination via EHR system
Documentation EHR system
Psychosoc. counseling required?
Practice service available?
Consultation (internal)
Coordination
Documentation EHR system
Rehabilitation required?
Consil
Coordination
Teleconsil
Documentation EHR system
Application for rehabilitation
Occupational medicine required?
Distribution of the questionnaire & coordination
Consil/ Case conference required?
Teleconsultation/ case conference
Documentation EHR system
Documentation EHR system
Neuroeducation (external)
Info in written form
Advisement (external)
Occupational health assessment
Positive exposure pattern & treatment suggestion
Advisement & support
if demanded
Application for rehabilitation
Documentation EHR system
Company Physician available?
Checklist self-assessment
Realization of therapy (by company physician)
