# Supplementary material for: Complex intervention for MS patients in Germany – can it be cost-saving for the society?
Source: Eur J Health Econ. 2026 Mar 4;27(7):1549–61. doi: 10.1007/s10198-026-01907-6 (PMC13619654; doi:10.1007/s10198-026-01907-6)
Supplement: Supplementary file 2 [file 10198_2026_1907_MOESM1_ESM.docx]

# 1 Attachment 1

MC-Simulation: Acronyms

| Acronym | Content |
| --- | --- |
| nP | Number of patients per practice |
| cP | Minute rate physician |
| cA | Minute rate for medical assistant |
| tPP | Project preparation (physician time) |
| tPA | Project preparation (medical assistant) |
| tCP | Quarterly consultation (physician time) |
| tCA | Quarterly consultation and coordination (medical assistant) |
| tAP | Functional assessments (physician time) |
| tAA | Functional assessments (medical assistant) |
| tNPP | Neuroeducation training (physician time) |
| tNP | Neuroeducation (physician time per session) |
| tNA | Neuroeducation (medical assistant l time per session) |
| tSPA | Counselling training (medical assistant time) |
| tSA | Counselling (medical assistant time) |
| tTP | Tele-consultation (physician time) |
| tMP | Case conference (physician time) |
| nPN | Number of patients per neuro-education module |
| nT | Number of tele-consultations per patient per year |
| nM | Number of case conferences per patient per year |

MC-Simulation – Model

| Output | Content | Equation |
| --- | --- | --- |
| N_nPr | Number of patients per practice | if(round(nP;0)<1;1;round(nP;0)) |
| C_PPr | Project preparation costs per practice (physician) | if(tPP<=120;120;if(tPP>960;960;tPP))*cP |
| C_PAr | Project preparation costs per practice (medical assistant) | if(tPA<=120;120;if(tPA>960;960;tPA))*cA |
| C_Pr | Project preparation costs per practice (total) | C_PPr+C_PAr |
| C_CPr | Costs per quarterly consultation (physician) | if(tCP<=5;5;if(tCP>60;60;tCP))*cP |
| C_CAr | Costs per quarterly consultation and coordination (medical assistant) | if(tCA>80;80;tCA)*cA |
| C_Cr | Costs per quarterly consultation and coordination (total) | C_CPr+C_CAr |
| C_APr | Costs per functional assessment (physician) | tAP*cP |
| C_AAr | Costs per functional assessment (physician) | if(tAA<15;15;if(tAA>100;100;tAA))*cA |
| C_Ar | Costs per functional assessment (total) | C_APr+C_AAr |
| C_NPPr | Costs of neuro-education training per practice (physician) | (if(tNPP<480;480;if(tNPP>1440;1440;tNPP)))*cP |
| C_NPr | Costs of neuro-education per session (physician) | (if(tNP<90;90;if(tNP>240;240;tNP)))*cP |
| C_NAr | Costs of neuro-education per session (medical assistant) | tNA*cA |
| C_Nr | Costs of neuro-education per session (total) | C_NPr+C_NAr |
| C_SPAr | Costs of training for counselling (medical assistant) | (if(tSPA<240;240;if(tSPA >480;480;tSPA)))*cA |
| C_SAr | Costs per counselling session (medical assistant) | if(tSA> 120;120;tSA)*cA |
| C_TPr | Costs per tele-consultation (physician) | tTP*cP |
| C_MPr | Costs per case conference (physician) | tMP*cP |
| R_P | Costs of project preparation per patient | C_Pr/N_nPr |
| R_C | Costs of quarterly consultation per patient & year | C_Cr*4 |
| R_A | Costs of functional assessments per patient & year | C_Ar*2 |
| R_NP | Costs of neuro-education training per patient & year | C_NPPr/N_nPr |
| R_N | Costs of neuro-education per patient per year | (C_Nr*7)/(if(N_nPr<=8;N_nPr;round(nPN-0,5))) |
| R_S | Costs of counselling training per patient per year | C_SPAr/N_nPr+C_SAr |
| R_T | Costs of teleconsultation per patient per year | C_TPr*round(nT-0,5) |
| R_M | Costs of case conference per patient per year | C_MPr*round(nM-0,5) |
| R | Total costs of care per patient per year | R_P+R_C+R_A+R_NP+R_N+R_S+R_T +R_M |

MC-Simulation Inputs

| Input | Distribution | Parameters | | | | | | | Unit |
| --- | --- | --- | --- | --- | --- | --- | --- | --- | --- |
| nP | Lognormal | Location: 2.5434 | | | Scale: 1.0222 | | | | number per year |
| cP | Fixed | 1.83 | | | | | | | € / min |
| cA | Fixed | 0.59 | | | | | | | € / min |
| tPP | Weilbull | Form:  1.4448 | | Scale: 305.808 | | Threshold: 43.9329 | | | min |
| tPA | Lognormal | Location: 5.9056 | | | Scale: 1.0659 | | | | min |
| tCP | Lognormal | Location: 3.1064 | | | Scale: 0.48417 | | | | min |
| tCA | Lognormal | Location: 2.9506 | | | Scale: 0.7013 | | | | min |
| tAP | Normal | Location: 15.9546 | Scale: 10.7281 | | Min:  2 | | | Max:  - | min |
| tAA | Lognormal | Location: 3.4002 | | | Scale: 0.6002 | | | | min |
| tNPP | Lognormal | Location: 6.2431 | | | Scale: 0.69284 | | | | min |
| tNP | Weibull | Form:  2.7113 | | Scale: 221.281 | | Threshold:  -51.0185 | | | min |
| tNA | Triangular | Min: 10 | | Median: 15 | | | Max: 240 | | min |
| tSPA | Lognormal | Location: 5. 1671 | | | Scale: 0.65507 | | | | min |
| tSA | Lognormal | Location: 3.5729 | | | Scale: 0.7519 | | | | min |
| tTP | Triangular | Min: 10 | | Median: 15 | | | Max: 20 | | min |
| tMP | Triangular | Min: 10 | | Median: 15 | | | Max: 20 | | min |
| nPn | Uniform | Min:4 | | | Max:8 | | | | number per year |
| nT | Uniform | Min:0 | | | Max:4 | | | | number per year |
| nM | Uniform | Min:0 | | | Max:2 | | | | number per year |

# 2 Attachment 2

Flowchart
